# The Impact of MS-Related Cognitive Fatigue on Future Brain Parenchymal Loss and Relapse: A 17-Month Follow-up Study

**DOI:** 10.3389/fneur.2016.00155

**Published:** 2016-09-21

**Authors:** Carina Sander, Paul Eling, Katrin Hanken, Jan Klein, Andreas Kastrup, Helmut Hildebrandt

**Affiliations:** ^1^Department of Psychology, University of Oldenburg, Oldenburg, Germany; ^2^Rehazentrum Wilhelmshaven, Wilhelmshaven, Germany; ^3^Donders Institute for Brain, Cognition and Behaviour, Radboud University, Nijmegen, Netherlands; ^4^Department of Neurology, Klinikum Bremen-Ost, Bremen, Germany; ^5^Fraunhofer MEVIS Institute for Medical Image Computing, Bremen, Germany

**Keywords:** multiple sclerosis, cognitive fatigue, MRI, brain atrophy, diffusion tensor imaging, disease progression, follow-up study

## Abstract

**Background:**

Fatigue is a disabling syndrome in multiple sclerosis (MS), which may be associated with inflammation and faster disease progression.

**Objective:**

To analyze the significance of cognitive fatigue for subsequent disease progression.

**Method:**

We followed 46 MS patients and 14 healthy controls in a study over 17 months. At the beginning (t1) and at the end (t2) of the study participants scored their fatigue, performed the Multiple Sclerosis Functional Composite and received MRI scanning, encompassing MPR T1, FLAIR, and DTI sequences. At t1, MS patients were divided into those with and those without cognitive fatigue (cut-off score for moderate cognitive fatigue of the Fatigue Scale for Motor and Cognition). We calculated ANCOVAs for repeated measurement to analyze the relevance of cognitive fatigue status for the number of relapses and for MRI parameters.

**Results:**

At t1, but not at t2, patients with cognitive fatigue showed increased axial and radial diffusivity of corpus callosum fibers. At t2, these patients showed significantly more loss of brain parenchyma and greater enlargement of lateral ventricles. Moreover, they developed more relapses, but there was no difference in lesion load or in performance deterioration. Additional analyses showed that only cognitive fatigue but not a more general score for fatigue (Fatigue Severity Scale) had an impact on the worsening of the disease status.

**Conclusion:**

Patients with cognitive fatigue may develop more brain atrophy and relapses during the next 17 months than patients without cognitive fatigue. Hence, experiencing cognitive fatigue might indicate more aggressive inflammatory processes and subsequent neurodegeneration.

## Introduction

Fatigue is a common disabling syndrome in multiple sclerosis (MS), which has been related to increased inflammation and faster disease progression (1). Recently, various authors proposed that fatigue may denote increased levels of proinflammatory cytokines and, therefore, an increased risk of T-cells invading the central nervous system (2–4). Previous studies examining this relation between fatigue and disease progression revealed inconsistent results. One study followed 11 MS patients for 3 months, performing serial MRI scans every 4 weeks (5). Gadolinium-enhancing lesions were unrelated to fatigue, arguing against a prognostic value of fatigue. A recent study on 127 patients with a clinically isolated syndrome revealed that patients with fatigue have a significantly higher chance to convert to MS (according to the McDonald criteria) than patients without fatigue (6), arguing for a prognostic value of fatigue.

Three studies analyzed the relation between fatigue and brain atrophy. One relied on retrospective scores of fatigue levels during the last 3 months and revealed no significant relation between fatigue and brain atrophy (7). Two studies used a prospective design and examined changes in brain atrophy over more than 4 years (8, 9). Both studies found that MS patients with fatigue suffered subsequently from enhanced brain atrophy.

Comparing these five studies, all used different fatigue measures and none focused on cognitive fatigue. Fatigue is a multi-componential symptom, which may encompass impairment related aspects [sometimes termed “fatigability” (1)] and the subjective experience of inflammation [a feature of sickness behavior (10)]. This distinction partly reflects the differentiation between motor fatigue, which seems to be related to motor impairment, and cognitive fatigue, which is a feeling independent of prior effort. Traditional scales, e.g., the Fatigue Severity Scale (FSS), used in most studies on fatigue and relapses or disease progression (11–13), do not differentiate between motor fatigue and cognitive fatigue. To study whether fatigue as feature of sickness behavior may indicate increased inflammation and more severe disease progression, scales assessing cognitive fatigue specifically should be used.

Neuropathological processes in MS are not restricted to gadolinium-enhancing lesions, but may also involve subclinical activation of glia cells, low level inflammation, and Wallerian degeneration. They might also be accompanied by partial remyelination of axons. The analysis of such processes requires quantified MRI analysis, focusing on changes in molecular diffusion as well as volumetry of brain parenchyma and the ventricles. Diffusion tensor imaging (DTI) offers the opportunity to study demyelination in normal appearing white matter. The corpus callosum, being composed of densely packed nerve fibers, seems to be well suited for DTI-analysis of disease progression in MS. For instance, Harrison et al. showed that radial diffusivity, calculated for the corpus callosum, is very sensitive to disease-related changes compared to other MRI measures (14). Moreover, changes in radial diffusivity can be well determined in small patient groups already after 1 or 2 years. Other studies have shown that brain and ventricle volumetry offer the possibility to measure longitudinal changes in MS patients (15).

In a prospective study, we examined the relation between cognitive fatigue and disease progression in MS patients, which were assessed twice with an interval of 17 months. We hypothesized that baseline cognitive fatigue levels might influence neurodegeneration, measured by changes in brain volume, and subclinical inflammation, as measured by axial and radial diffusivity of the corpus callosum.

## Materials and Methods

### Participants

Based on the power calculation of Harrison et al. (14), we included 46 MS patients and 14 healthy controls. Patients were recruited from self-help groups or they were patients of neurologists in Bremen and surroundings. They were aged between 18 and 46 years, diagnosed with a relapsing-remitting or secondary progressive MS according to the McDonald Criteria (16) and had an Expanded Disability Status Scale (17) of 0-6.5. Patients with a relapse during the 4 weeks before the investigation were excluded.

Age, education, and gender matched healthy controls were mainly recruited from the nursing staff of the Klinikum Bremen-Ost as well as from the staff of the Fraunhofer MEVIS Institute. Participants confirmed not to have any neurological or psychiatric disorders.

All participants gave their informed consent. The Ethical Board of the Bremer Physicians Society approved the study.

### Assessment of Clinical Data

To assess MS-related fatigue, the Fatigue Scale for Motor and Cognitive Functions (FSMC) was applied. The FSMC is composed of 20 items and evaluates two main components of fatigue, namely motor and cognitive fatigue (18). Additionally, we applied the FSS (12). Beck’s Depression Inventory (BDI) was applied to assess depressive mood, but items that assess somatic concerns associated with depression were excluded, because of their overlap with experiencing fatigue (19, 20). As a measure of the clinical status (leg and arm function as well as cognitive functioning) we included the Multiple Sclerosis Functional Composite (MSFC). The subtest Paced Auditory Serial Addition Test (PASAT) of the MSFC was included to measure cognitive functioning (21). Participants were assessed twice (t1, t2) with a delay of 17 months. Finally, at t2, we asked patients about the occurrence of relapses during the evaluation period.

### Image Acquisition

A 3.0T scanner (Siemens Skyra, Erlangen, Germany) was used to attain magnetic resonance images of the brain. By using 64 non-collinear orientations in the axial plane [image resolution 2 mm × 2 mm × 2 mm, ~55 slices, TR = 7600, TE = 90, number of excitations (NEX) = 1, b-value = 1000, scanning time ~9 min], diffusion MRI images were obtained. To receive T1-weighted images (TR = 1900, TE = 2.43) and FLAIR-weighted images (TR = 5000, TE = 388), an isotropic resolution of 1 mm × 1 mm × 1 mm was used. These were also used to investigate brain atrophy measures and lesion segmentations.

### Measurement of Lesion Load, Brain Atrophy, and Corpus Callosum

Analysis of the MRI data was performed using the NeuroQLab3.531 software package (Fraunhofer MEVIS, Bremen, Germany). We calculated lesion load using the FLAIR images. For calculation of gray and white matter volume (total brain volume, TBV), the brain parenchymal fraction (BPF) and the volume of the lateral, third, and fourth ventricles T1-weighted images were used.

T1-weighted images were also utilized to compute the corpus callosum index (CCI). For regional measurements, we split the corpus callosum into three segments (anterior, middle, posterior), and divided each by the anterior–posterior diameter of the corpus callosum [see Yaldizli et al. (22)].

### Fiber Tractography and Quantification

The advection–diffusion-based algorithm was used for tracking fibers (23). For the corpus callosum, the seed region was defined in a sagittally oriented slice located above the lateral ventricles. Two parts of the corpus callosum were tracked separately, in particular the frontal part (from the frontal tip to vertex) and the posterior part (from vertex to back tip). To quantify each part, two crop ROIs were defined in sagittally oriented slices at the points where fibers run caudally to merge with pyramidal tracts. Mean values of axial and radial diffusivity were calculated for each participant. A detailed description of the quantification method can be found in Klein et al. (24).

### Statistical Analysis

We split the MS group in a group without cognitive fatigue and a group with cognitive fatigue, according to the Manual of the FSMC, using 28 as cut-off score for moderate cognitive fatigue (18).

To compare the groups at t1, parametrical and non-parametrical tests were used depending on the data-distributions, analyzed with the Kolmogorov–Smirnov test.

We used a repeated measurement ANCOVA to analyze the impact of cognitive fatigue on MRI parameters and performance scores. TIME (t1 and t2) served as within-subject variable and GROUP (healthy controls, MS patients with and without cognitive fatigue) as between subject variable. We included AGE as a covariate since age is associated with a decline of brain volume and ventricle size. The DTI measures axial and radial diffusivity were used as within subject variable in a repeated measurement ANCOVA. *Post hoc* analysis of interaction effects was performed with difference scores for significant MRI variables. For *post hoc* testing, we used the Bonferroni correction for multiple comparisons.

To check for influences of the clinical phenotype of MS, the same analyses were also performed separately for relapsing-remitting MS patients and for secondary progressive MS patients.

To get an idea of the effects of cognitive fatigue in comparison to those of moderate motor fatigue or moderate global fatigue [FSS, which has been used in previous studies (11–13)], we repeated the above-mentioned analyses, splitting MS patients in a group with a motor fatigue score less than 27 and a group with a score of 27 and higher (18), as well as in a group with an FSS score of less than 5 and a group with a score of 5 and higher (12). At the same time, it should be noted that our study was not designed for and does not allow a direct comparison between different aspects of fatigue.

## Results

### Groups Characteristics at t1

After drop out of four patients from the MS group and one participant from the control group at t2, the data of 42 patients and 13 controls were analyzed. Reasons for drop out were a pregnancy, familial problems, relocation without actualization of the address (*n* = 2) and a withdrawal of the agreement to participate. Baseline characteristics of the patients and the control group are shown in Table 1.

**Table 1:** **Baseline characteristics and FSS, EDSS, BDI, and FSMC (total, cognition, and motor subscales) outcome for the healthy controls and the two patient groups**.

	Control group	No cognitive fatigue	Cognitive fatigue
Sex (females/males), *n* (%)	9/4 (69.2%)	9/5 (64.3)	18/10 (64.3)
Clinical phenotype *n* (%)			
Relapsing-remitting	N/A	12 (85.7)	18 (64.3)
Secondary progressive		2 (14.3)	10 (35.7)
No medication/interferons/escalated medications (%)^a^	100/0/0	15.4/53.8/30.8	46.7/40.0/13.3
Age (years) (mean, SD)^a^	48.6 (5.0)	42.8 (12.8)	50.5 (8.8)
Education (years) (median, R)^a^	13.0 (5.0)	12.5 (3)	10.0 (5.0)
FSS (mean, SD)^a^	19 (9.87)	29 (10.25)	46 (10.64)
FSMC cognition (median, R)	17.0 (26)	21.0 (14)	36.5 (21)
FSMC motor (mean, SD)	15.5 (6.5)	26.21 (6.9)	38.93 (6.1)
Relapses during the evaluation period^a^	N/A	1 out of 14	13 out of 28
Relapsing-remitting	N/A	2 out of 12	9 out of 18
Secondary progressive	N/A	1 out of 2	2 out of 10

*FSS, Fatigue Severity Scale; FSMC, Fatigue Scale for Motor and Cognitive Functions; MS, multiple sclerosis*.

*^a^Significant differences between the MS groups computed as described above*.

The comparison of MS patients with and without cognitive fatigue revealed no significant differences in gender ratio. Furthermore no differences were found in terms of the number of patients suffering from progressive and non-progressive types of MS as well as for the EDSS score and the total lesion load. The number of MS patients, receiving disease-modifying drugs, divided into those with classical interferons and those receiving escalating treatments with Natalizumab or Fingolimod, failed to reach significance [χ(2) = 4.827, *p* = 0.089] (see Table 1).

The two patients groups differed in age [*F*(25.437) = −2.378, *p* = 0.026] and BDI score [*F*(1) = 6.675, *p* = 0.010], FSS score [*F*(1) = 15.752, *p* < 0.001] (see Table 1).

### Cognitive Fatigue Status at t1 and Disease Progression

Table 2 gives an overview of the following results.

**Table 2:** **Mean and SD as well as the main and interaction effects given by the ANCOVA for repeated measurements controlling for AGE for the group with and without cognitive fatigue as well as for the *control group***.

	Cognitive fatigue (*n* = 28)		No cognitive fatigue (*n* = 14)		Healthy controls (*n* = 13)		Sign.
	T1	T2	T1	T2	T1	T2	
	Mean (SD)	Mean (SD)	Mean (SD)	Mean (SD)	Mean (SD)	Mean (SD)	
BDI	7.39 (6.53)	5.39 (6.34)	2.71 (2.53)	2.00 (1.92)	3.23 (4.44)	1.92 (2.22)	a, b
EDSS	4.14 (1.52)	3.70 (1.46)	2.63 (2.12)	3.42 (2.00)	N/A	N/A	b
MSFC	−0.15 (0.53)	−0.02 (0.50)	−0.01 (0.48)	0.09 (0.60)	0.47 (0.41)	0.57 (0.34)	a
PASAT	43.67 (13.11)	47.89 (9.10)	45.07 (8.41)	45.93 (11.26)	47.77 (10.32)	51.54 (11.23)	
Lesion load	6.94 (14.86)	7.03 (15.07)	3.25 (2.71)	3.21 (2.63)	0.03 (0.08)	0.07 (0.18)	b
TBV	1221.1 (125.01)	1199.8 (120.43)	1191.2 (118.33)	1190.0 (117.86)	1280.9 (72.55)	1275.3 (75.80)	c
BPF	0.81 (0.04)	0.79 (0.04)	0.82 (0.04)	0.81 (0.04)	0.85 (0.15)	0.84 (0.02)	a
Lateral ventricles	40.24 (29.29)	41.60 (29.75)	33.61 (26.33)	33.26 (26.09)	16.78 (6.43)	16.88 (6.80)	a, c
Volume of third and fourth ventricle	4.13 (1.40)	4.21 (1.40)	2.99 (1.46)	3.24 (1.39)	2.72 (1.27)	2.85 (1.31)	a, b
CCI	0.33 (0.08)	0.36 (0.07)	0.33 (0.70)	0.37 (0.07)	0.43 (0.05)	0.45 (0.04)	a
Axial and radial diffusivity of the corpus callosum	0.0013 (0.0001)	0.0014 (0.0002)	0.0013 (0.0001)	0.0013 (0.0002)	0.0012 (0.0000)	0.0012 (0.0001)	a, c

*BDI, Beck’s Depression Inventory; BPF, brain parenchymal fraction; CCI, corpus callosum index; EDSS, Expanded Disability Status Scale; FSMC, Multiple Sclerosis Functional Composite; MS, multiple sclerosis; PASAT, Paced Auditory Serial Addition Test; TBV, total brain volume*.

*Significant difference (*p* < 0.05) for a: main factor GROUP, b: significant main factor TIME, c: significant interaction group × time*.

Significantly more patients with cognitive fatigue suffered relapses during the 17 months interval than patients without cognitive fatigue [χ(1) = 5.027, *p* = 0.018].

For the EDSS only a significant main effect for TIME [*F*(1,37) = 16.435, *p* < 0.001] and an interaction effect between TIME and AGE [*F*(1,37) = 16.327, *p* < 0.001] were found.

The analysis of the MSFC data revealed a significant main effect for Group [*F*(2,48) = 8.25, *p* = 0.001], but no significant main effect of TIME and no interaction effect. The patient group had a significantly lower MSFC score than the control group but there was no significant difference between the patient groups, as *post hoc* analysis revealed. A separate analysis of the Subtest PASAT of the MSFC did reveal a main effect for AGE [*F*(1,50) = 4.402, *p* = 0.041] but no significant other main or interaction effects.

A main effect for TIME was found for the mental items of the BDI [*F*(1,51) = 6.109, *p* = 0.017]. The BDI score of the total group decreased significantly over time. A main effect of GROUP was found [*F* (2,51) = 4.023, *p* = 0.024] too, but no interaction with TIME. *Post hoc* analysis revealed that patients with cognitive fatigue had a significantly higher score for the mental items of the BDI than patients without cognitive fatigue and the control group. The scores of the patient group without fatigue and the control group were not significantly different.

Concerning the MRI parameters, significant main effects for GROUP were found for the axial and radial diffusivity of the corpus callosum [*F*(2,51) = 6.67, *p* = 0.003], the BPF [*F*(2,51) = 5.85, *p* = 0.005], the CCI [*F*(2,51) = 11.41, *p* = 0.001], the lateral ventricles [*F*(2,51) = 4.12, *p* = 0.022] and the third and fourth ventricle [*F*(2,51) = 4.48, *p* = 0.016]. *Post hoc* analyses revealed significant differences between the patient groups and the control group, but not between the patient groups. The patient group had higher axial and radial diffusivity of the corpus callosum, a lower BPF, a lower CCI and higher volumes of the lateral, third, and fourth ventricles compared to the control group.

Significant main effects for TIME were found for the third and fourth ventricle volume [*F*(1,50) = 4.125, *p* = 0.048]. The volume of the ventricles increased in all three groups.

Significant AGE effects were obtained for the parameter TBV [*F*(1,51) = 5.137, *p* = 0.028].

**Figure 1:**
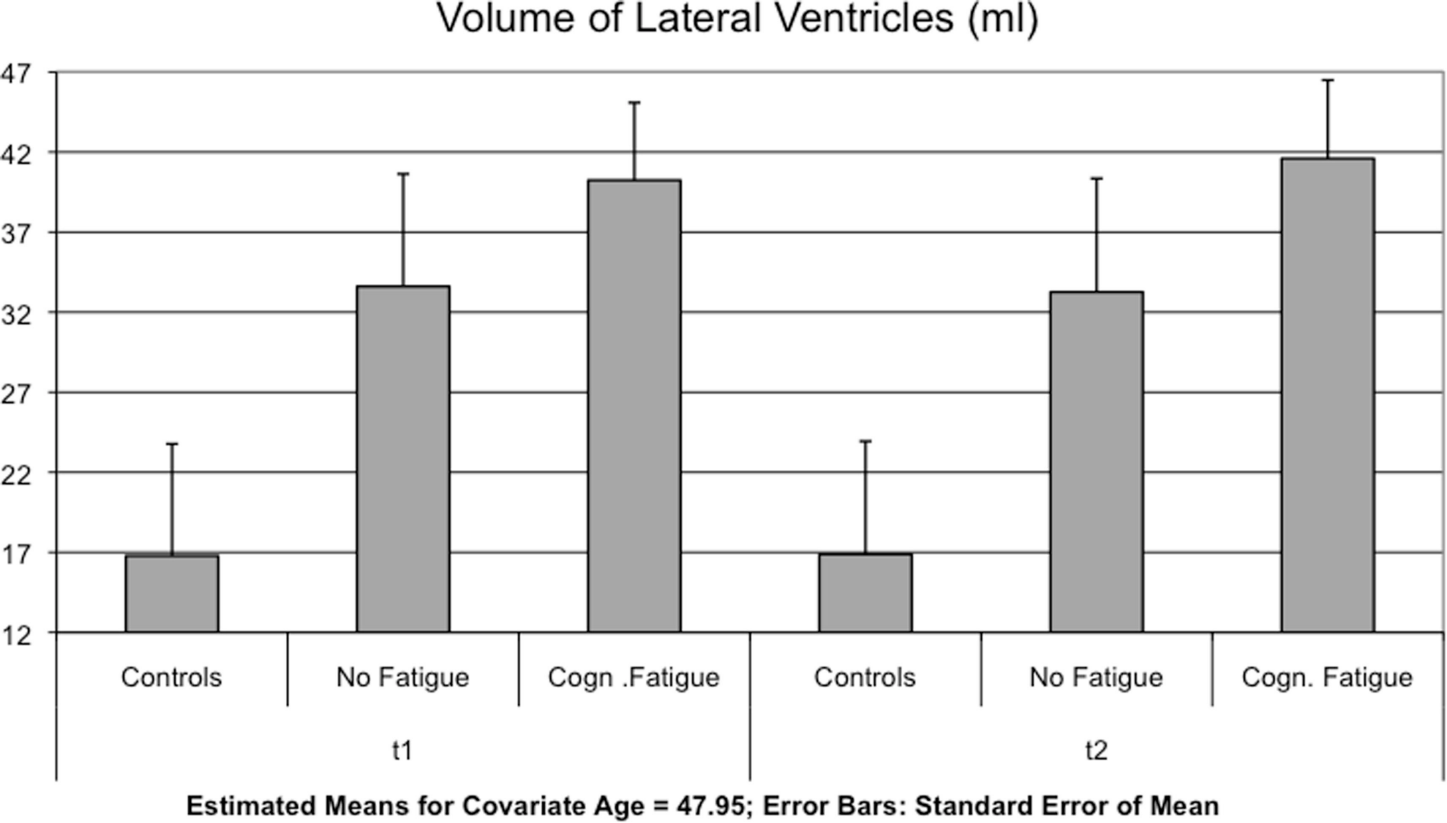
**The mean of the volume of the lateral ventricles is shown for each group and time point t1 and after 17 months (t2)**.

**Figure 2:**
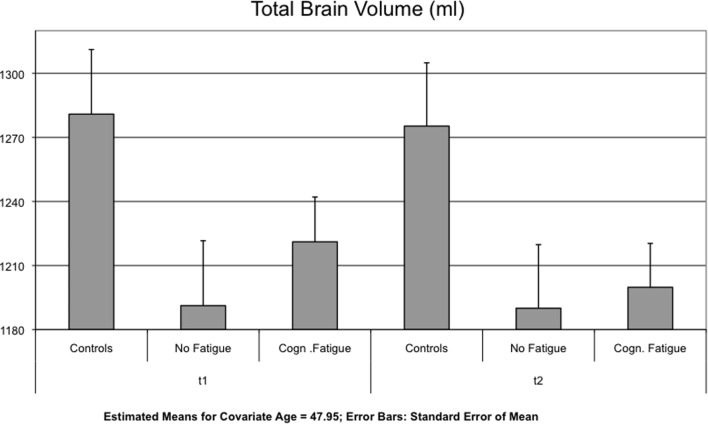
**Mean of the total brain volume (TBV) is displayed for each group and time point t1 and after 17 months (t2)**.

Interaction effects between GROUP and TIME were found for the lateral ventricles [*F*(2,51) = 4.123, *p* = 0.022] (Figure 1), the TBV [*F*(2,51) = 5.328, *p* = 0.008] (Figure 2) and the axial and radial diffusivity of the corpus callosum [*F*(2,51) = 3.722, *p* = 0.031].

Bonferroni corrected multiple comparisons among groups of changes in lateral ventricle volume revealed a significant difference between patients with and without cognitive fatigue (1.37 ± 1.98 vs. 0.37± 2.01 ml, *p* = 0.019). No significant differences were found between the MS group without fatigue and the control group (*p* < 0.999) and the MS group with fatigue and the control group (*p* = 0.143) (see Table 3). Using the less conservative LSD *post hoc* test, the difference between the control group and the MS patient group with cognitive fatigue was significant for the lateral ventricles (*p* = 0.048), as well as for the TBV (*p* = 0.021).

**Table 3:** **Mean and SD of the differences between t1 and t2 given for variables with the significant *interaction effects in the ANCOVA analysis***.

	Control group		No cognitive fatigue		Cognitive fatigue	
	Mean	SD	Mean	SD	Mean	SD
TBV^a^^,^^b^	5.59	14.11	1.50	27.84	21.24	16.78
Lateral ventricles^a^^,^^b^	−0.10	1.42	−0.37	2.01	−1.37	1.98
Axial and radial diffusivity of the corpus callosum^b^^,^^c^	0.0000360	0.0000670	0.0000416	0.0000702	0.0000202	0.0000872

*TBV, total brain volume*.

*^a^Significant differences between both MS groups using Bonferroni post hoc test*.

*^b^Significant differences between control group and MS group with fatigue using LSD post hoc test*.

*^c^Significant differences between both MS groups using LSD post hoc test*.

Concerning the change in TBV, *post hoc* testing revealed a significant difference between the MS group with cognitive fatigue [−21.24 ± 16.78 ml] and the group without cognitive fatigue [−0.37 ± 2.01 ml, *p* = 0.010]. No significant differences were found for the comparison of the two MS groups with the control group (see Table 3).

There were no significant differences between all three groups for the axial and radial diffusivity of the corpus callosum using the Bonferroni correction (see Table 3). But the less conservative LSD *post hoc* tests revealed significant differences between both MS groups concerning the axial and radial diffusivity of the corpus callosum (*p* = 0.038) and between the control group and the MS group with fatigue (*p* = 0.038).

#### Comparing MS Patients with and without Cognitive Fatigue According to Their Clinical Phenotype

Table 4 gives an overview of the following results.

**Table 4:** **Mean and SD and the main and interaction effects given by the ANCOVA for repeated measurements controlling for AGE for the group with and without cognitive fatigue separated according to the progress form of MS**.

	RR cognitive fatigue (*n* = 18)		RR no cognitive fatigue (*n* = 12)		Sign.	ScP cognitive fatigue (*n* = 10)		ScP no cognitive fatigue (*n* = 2)	
	T1	T2	T1	T2		T1	T2	T1	T2
	Mean (SD)	Mean (SD)	Mean (SD)	Mean (SD)		Mean (SD)	Mean (SD)	Mean (SD)	Mean (SD)
BDI	7.39 (7.09)	5.38 (7.27)	3.00 (2.6)	2.08 (2.07)		7.40 (5.74)	5.60 (4.55)	1.00 (1.41)	1.50 (0.71)
EDSS	3.64 (1.41)	3.69 (1.63)	2.15 (1.94)	3.35 (2.12)		5.05 (1.34)	3.70 (1.18)	5.00 (1.41)	3.75 (1.77)
MSFC	−0.06 (0.50)	0.11 (0.43)	0.06 (0.48)	0.18 (0.59)	a	−0.33 (0.60)	−0.26 (0.56)	−0.38 (0.40)	−0.43 (0.52)
PASAT	44.47 (13.27)	48.35 (9.11)	45.08 (8.78)	46.08 (11.62)		42.30 (13.43)	47.10 (9.53)	45.00 (8.49)	45.00 (12.73)
Lesion load	4.07 (7.15)	4.11 (7.22)	2.93 (2.77)	2.87 (2.64)		12.10 (22.80)	12.28 (23.14)	5.11 (1.68)	5.28 (1.83)
TBV	1223.38 (147.16)	1199.35 (140.90)	1222.29 (107.99)	1219.26 (108.11)	c	1192.42 (72.75)	1176.20 (75.00)	1141.25 (206.12)	1148.95 (208.67)
BPF	0.83 (0.04)	0.81 (0.04)	0.82 (0.04)	0.81 (0.05)	a	0.78 (0.03)	0.77 (0.03)	0.82 (0.02)	0.81 (0.02)
Lateral ventricles	28.97 (19.28)	30.41 (20.00)	31.37 (28.50)	31.59 (28.32)		62.26 (32.87)	63.51 (33.41)	37.65 (3.07)	33.76 (4.42)
Volume of third and fourth ventricle	3.68 (1.17)	3.74 (1.16)	2.89 (1.53)	3.17 (1.43)		4.93 (1.46)	5.07 (1.45)	3.63 (1.06)	3.67 (1.47)
CCI	0.36 (0.06)	0.39 (0.05)	0.33 (0.06)	0.38 (0.07)	a	0.26 (0.04)	0.29 (0.05)	0.32 (0.06)	0.31 (0.08)
Axial and radial diffusivity of the corpus callosum	0.0013 (0.0001)	0.0012 (0.0001)	0.0013 (0.0001)	0.0013 (0.0002)	a	0.0015 (0.0001)	0.0014 (0.0002)	0.0014 (0.0001)	0.0014 (0.0001)

*BDI, Beck’s Depression Inventory; BPF, brain parenchymal fraction; CCI, corpus callosum index; EDSS, Expanded Disability Status Scale; FSMC, Multiple Sclerosis Functional Composite; MS, multiple sclerosis; PASAT, Paced Auditory Serial Addition Test; TBV, total brain volume; RR, relapsing-remitting MS; ScP, secondary progressive MS*.

*Significant difference (*p* < 0.05) for a: main factor GROUP, b: significant main factor TIME, c: significant interaction group × time*.

Analyzing the data only for relapsing-remitting MS patients (*n* = 30), the MSFC showed a main effect of GROUP [*F*(2,37) = 7.468, *p* = 0.002], but we did not find any other significant group effects for clinical variables (Table 4 gives an overview of the results). *Post hoc* analyses revealed significant differences between the patient groups and the control group, but not between the patient groups.

Analysis of the MRI parameters revealed main effects for GROUP for the BPF [*F*(2,39) = 4.029, *p* = 0.026], the CCI [*F*(2,39) = 9.355, *p* < 0.001] and the DTI measures [*F*(2,39) = 6.331, *p* = 0.004], all pointing to a higher impairment of the patient group compared to the control group.

Main effects for TIME were found for the CCI [*F*(1,39) = 12.799, *p* = 0.001] and the third and fourth ventricle [*F*(1,39) = 7.188, *p* = 0.011].

Interaction effects between GROUP and TIME were found for the TBV [*F*(2,39) = 4.100, *p* = 0.024], but there were no significant differences between all three groups for the TBV using the Bonferroni corrected multiple comparisons as well as the less conservative LSD *post hoc* test.

None of the other clinical or MRI variables showed an interaction effect of GROUP and TIME.

Only two of the 12 patients with a secondary chronic disease course did not suffer from at least moderate fatigue. Therefore, the statistical comparison between patients with and without fatigue was canceled.

### Motor or Global Fatigue Status at t1 and Disease Progression

#### Comparing MS Patients with and without Motor Fatigue

There was no significant difference in relapse rate comparing patients with and without motor fatigue.

Table 5 gives an overview of the following results.

**Table 5:** **Mean and SD and the main and interaction effects given by the ANCOVA for repeated measurements controlling for AGE for the group with and without motor fatigue**.

	Motor fatigue (*n* = 32)		No motor fatigue (*n* = 9)		Healthy controls (*n* = 13)		Sign.
	T1	T2	T1	T2	T1	T2	
	Mean (SD)	Mean (SD)	Mean (SD)	Mean (SD)	Mean (SD)	Mean (SD)	
BDI	6.73 (6.26)	4.88 (5.99)	2.56 (2.79)	2.00 (2.06)	3.23 (4.44)	1.92 (2.22)	b
EDSS	4.10 (1.59)	3.74 (1.44)	2.28 (2.02)	3.17 (2.17)	N/A	N/A	a, b
MSFC	−0.13 (0.54)	0.013 (0.53)	−0.00 (0.48)	0.04 (0.58)	0.47 (0.41)	0.57 (0.34)	a
PASAT	43.94 (12.32)	48.38 (8.99)	44.89 (9.35)	43.11 (11.91)	47.77 (10.32)	51.54 (11.23)	
Lesion load	6.42 (13.74)	6.50 (13.93)	3.11 (2.91)	3.03 (2.75)	0.03 (0.08)	0.07 (0.18)	
TBV	1206.32 (124.42)	1190.68 (121.67)	1231.833 (114.05)	1220.76 (109.06)	1280.9 (72.55)	1275.3 (75.80)	
BPF	0.81 (0.04)	0.80 (0.04)	0.82 (0.05)	0.80 (0.05)	0.85 (0.15)	0.84 (0.02)	a
Lateral ventricles	38.58 (27.79)	39.47 (28.36)	35.84 (31.82)	36.29 (31.48)	16.78 (6.43)	16.88 (6.80)	a
Volume of third and fourth ventricle	3.94 (1.39)	4.07 (1.38)	3.04 (1.76)	3.23 (1.61)	2.72 (1.27)	2.85 (1.31)	a
CCI	0.33 (0.07)	0.36 (0.07)	0.33 (0.06)	0.37 (0.07)	0.43 (0.05)	0.45 (0.04)	a, b
Axial and radial diffusivity of the corpus callosum	0.0013 (0.0001)	0.0013 (0.0001)	0.0013 (0.0001)	0.0013 (0.0001)	0.0012 (0.0000)	0.0012 (0.0001)	a

*BDI, Beck’s Depression Inventory; BPF, brain parenchymal fraction; CCI, corpus callosum index; EDSS, Expanded Disability Status Scale; FSMC, Multiple Sclerosis Functional Composite; MS, Multiple Sclerosis; PASAT, Paced Auditory Serial Addition Test; TBV, total brain volume*.

*Significant difference (*p* < 0.05) for a: main factor GROUP, b: significant main factor TIME, and c: significant interaction group × time*.

A main effect for the mental items of the BDI was found for TIME [*F*(1,51) = 4.754, *p* = 0.034], showing a decline of the BDI scores of the total group over time. Additionally, a main effect of TIME was found for the EDSS [*F*(1,37) = 17.594, *p* < 0.001] as well as a main effect of GROUP [*F*(2,37) = 6.418, *p* = 0.016] and an interaction effect between TIME and AGE [*F*(1,37) = 17.159, *p* < 0.001]. The patient group with motor fatigue had a higher EDSS than the patient group without motor fatigue.

A main effect for GROUP was found for the MSFC [*F*(2,48) = 8.253, *p* = 0.001]. The MSFC score was lower in patients without motor fatigue, compared to the patients with motor fatigue and also compared to the group of healthy controls, who had the highest scores.

The analyses on patients with and without motor fatigue also showed significant main effects for GROUP for the BPF [*F*(2,51) = 5.800, *p* = 0.0005], the lateral ventricles [*F*(2,51) = 3.591, *p* = 0.035], the third and fourth ventricles [*F*(2,51) = 3.643, *p* = 0.033], the CCI [*F*(2,51) = 11.444, *p* < 0.001] and the DTI measures [*F*(2,51) = 6.708, *p* = 0.003]. *Post hoc* analyses revealed significant differences between the patient groups and the control group, but not between the patient groups. The patient group had a lower BPF, a higher volume of the ventricles, a lower CCI, and a higher axial and radial diffusivity of the corpus callosum compared to the healthy controls.

Significant main effects for TIME were found for the CCI [*F*(1,51) = 13.086, *p* = 0.001]. No significant interaction effects between TIME and GROUP were found for any of the variables.

#### Comparing MS Patients with and without Fatigue Using the FSS

There was no significant difference in relapse rate between patients with and without fatigue according to the FSS.

Table 6 gives an overview of the following results.

**Table 6:** **Mean and SD and the main and interaction effects given by the ANCOVA for repeated measurements controlling for AGE for the group with and without fatigue according to the FSS**.

	Fatigue (*n* = 17)		No fatigue (*n* = 25)		Healthy controls (*n* = 13)		Sign.
	T1	T2	T1	T2	T1	T2	
	Mean (SD)	Mean (SD)	Mean (SD)	Mean (SD)	Mean (SD)	Mean (SD)	
BDI	7.29 (7.32)	6.41 (7.75)	4.84 (4.64)	2.8 (2.48)	3.23 (4.44)	1.92 (2.22)	
EDSS	4.56 (1.27)	3.91 (1.28)	3.04 (1.95)	3.39 (1.83)	N/A	N/A	a, b
MSFC	−0.18 (0.44)	−0.07 (0.34)	−0.06 (0.57)	0.07 (0.62)	0.47 (0.41)	0.57 (0.34)	
PASAT	42.50 (12.60)	47.13 (8.52)	45.20 (11.10)	47.28 (10.70)	47.77 (10.32)	51.54 (11.23)	
Lesion load	3.71 (2.57)	3.80 (2.61)	7.07 (15.77)	7.09 (15.99)	0.03 (0.08)	0.07 (0.18)	
TBV	1201.01 (109.05)	1178.80 (103.78)	1219.12 (130.77)	1209.59 (128.02)	1280.9 (72.55)	1275.3 (75.80)	
BPF	0.81 (0.04)	0.80 (0.04)	0.81 (0.04)	0.81 (0.04)	0.85 (0.15)	0.84 (0.02)	
Lateral ventricles	40.92 (24.68)	41.92 (25.03)	36.00 (30.87)	36.65 (31.25)	16.78 (6.43)	16.88 (6.80)	
Volume of third and fourth ventricle	4.22 (1.47)	4.31 (1.44)	3.43 (1.46)	3.61 (1.43)	2.72 (1.27)	2.85 (1.31)	
CCI	0.326 (0.067)	0.352 (0.067)	0.329 (0.068)	0.368 (0.071)	0.43 (0.05)	0.45 (0.04)	b
Axial and radial diffusivity of the corpus callosum	0.0013 (0.0001)	0.0013 (0.0001)	0.0013 (0.0001)	0.0013 (0.0002)	0.0012 (0.0000)	0.0012 (0.0001)	

*BDI, Beck’s Depression Inventory; BPF, brain parenchymal fraction; CCI, corpus callosum index; EDSS, Expanded Disability Status Scale; FSS, Fatigue Severity Scale; FSMC, Multiple Sclerosis Functional Composite; MS, multiple sclerosis; PASAT, Paced Auditory Serial Addition Test; TBV, total brain volume*.

*Significant difference (*p* < 0.05) for a: main factor GROUP, b: significant main factor TIME, and c: significant interaction group × time*.

The analyses also showed no main or interaction effects for the mental items of the BDI, BPF, TBV, lateral ventricle volume, third and fourth ventricle volume, lesion load, axial and radial diffusivity, and the MSFC, including the PASAT.

There was a main effect of TIME for the CCI [*F*(1,39) = 11.019, *p* = 0.002], which increased over time in all three groups. For the EDSS, we found a main effect of TIME [*F*(1,37) = 15.394, *p* < 0.001] as well as a main effect of GROUP [*F*(1,37) = 6.795, *p* = 0.013]. The EDSS score of the total group decreased over time. The EDSS score of the patients with fatigue was higher than the EDSS score of the patients without fatigue. An interaction effect between TIME and AGE [*F*(1,37) = 16.829, *p* < 0.001] was found too.

There was no interaction effect between GROUP and TIME for any of the analyzed variables.

## Discussion

The main findings show that patients with at least moderate cognitive fatigue had an increased axial and radial diffusivity in the corpus callosum, an increased volume of the lateral ventricle and a decreased brain volume (gray and white matter volume) after 17 months compared to a group of healthy controls and, more importantly, to a group of MS patients without cognitive fatigue.

These findings suggest that increased cognitive fatigue at t1 may be a prognostic marker for disease progression and this has not been shown in any previous study. Our findings underscore those of Runia et al. (6), showing that patients with clinically isolated syndrome suffering from fatigue have a greater risk to convert to clinically definite MS. It is noteworthy that the MS group with cognitive fatigue more often suffered from relapses during the interval than the group without cognitive fatigue (Table 1) and that this difference is significant.

Experiencing relatively high levels of fatigue, reflecting the subjective component of sickness behavior (2), may indicate more aggressive inflammatory processes at t1 leading to loss of brain volume at t2. Accordingly, changes from t1 to t2 were significant for lateral ventricle size and TBV for the MS patients with cognitive fatigue but not for lesion load, CCI, and BPF (although the absolute figures pointed in the right direction). These findings basically replicate findings from two prospective studies, but there are also relevant differences (8, 9). First, the interval in our study was 17 months, which is much shorter than in the two previous studies. Hence, according to our results the long-term significance of fatigue can be measured in a period that approximately matches the interval used for routine clinical scanning. Second, in contrast to other studies, we focused on TBV and lateral ventricle size. The latter is highly influenced by an increasing periventricular lesion load and the so-called Dawson fingers. Therefore, the size of the lateral ventricles should be a very sensitive marker for disease progression. TBV reflects brain volume directly, BPF as fraction of cranial volume. Changes in BPF, therefore, are influenced by two measurement errors, that of brain volume and of cranial volume. Because a standardized measure of brain volume is less important for longitudinal studies, in a longitudinal design TBV seems preferable; whereas in cross-sectional studies, BPF seems the better measurement.

Cognitive fatigue at t1 was related to increased axial and radial diffusivity in the corpus callosum. In a previous study, we also found increased values for axial and radial diffusivity in the corpus callosum of MS patients, but there we did not analyze its relation to fatigue (25). Increased radial diffusivity has been related to demyelination, allowing water molecules to move in vertical direction of myelinated axons. During inflammation, axial diffusivity is usually decreased, because of swelling and inflammation [see also Fink et al. (25)]. But in later stages axial diffusivity may increase indicating either recovery or Wallerian degeneration. Interpretation of increased axial diffusivity is, therefore, difficult. Here, we compared the MS groups with and without cognitive fatigue directly with a healthy control group and we found no threefold interaction between GROUP, TIME, and DIFFUSIVITY, but only an interaction between GROUP and TIME. MS patients without fatigue and healthy controls showed a similar increase in axial and radial diffusivity over time, whereas MS patients with fatigue started with higher axial and radial diffusivity scores but decreased in diffusivity over time. The higher axial and radial diffusivity at t1, therefore, seems to be the critical finding for the group with cognitive fatigue and not the lower score at t2 (as in the case of brain atrophy). The increase in diffusivity may, therefore, indicate increased demyelination at t1, which may have terminated at t2, where it could be measured as increased ventricle volume and loss of white and gray matter.

This interpretation seems to hold for MRI parameters, but not for performance measures. Three previous studies with more patients than in our study and focusing on the clinical relation between fatigue and disease progression yielded similar negative results for performance scores (11, 13, 26). One of these studies used a period similar to ours (18 months) (13), the other two a period of 1 (26) and 10 years (11).

Taken together, these findings indicate that MRI parameter may be more sensitive for assessing disease progression than variables assessing clinical functions.

The focus of this study was specifically on cognitive fatigue (see Introduction). To put our results into context, we re-analyzed the data by dividing the MS patient groups into those with at least moderate motor fatigue or moderate global fatigue according to the FSS and those without fatigue. Using these two other fatigue scores, we found several significant differences between the healthy control group and one or both MS groups concerning performance and MRI parameters, but no interaction effect between GROUP and TIME. This supports the idea that cognitive fatigue compared to other fatigue measures is especially sensitive for indicating more aggressive inflammatory processes at t1. At the same time, it should be noted that there is considerable overlap among patients with cognitive fatigue and with motor fatigue. Actually, the correlation between cognitive fatigue and motor fatigue at t1 was *r* = 0.81 and that between cognitive fatigue and the FSS score *r* = 0.77. Hence, to analyze the meaning of different fatigue measures in more detail larger patients groups will be necessary and regression analysis should be used.

Multiple sclerosis patients with and without fatigue differed from the control group on most MRI parameters, but they did not differ from each other. This finding fits earlier negative results on the relation of a more global atrophy marker and fatigue (3). More specific analyses of the relation between cognitive fatigue, as used in this study, and MRI parameters are rare, but a recent study of Wilting et al. (27) showed that cognitive fatigue might be related to atrophy of the thalamus and not to more global variables as brain parenchymal fraction, gray and white matter. The other significant finding from our study, arguing for a relation between age and brain atrophy, does not require additional discussion.

We found, as other studies did, that depressive mood did not increase over the 17-month period (28). Depressive mood may be linked to experiencing relapses and the feeling of losing control over the disease. Hence, mood may improve in patients in more stable disease conditions, whereas it may deteriorate in patients in instable disease conditions.

We tried to analyze whether clinical phenotype played a major role for progression. Unfortunately, the number of secondary chronic progressive patients was (especially for the group without fatigue) too small for statistical analysis. For the relapsing-remitting patients (*n* = 30), there still was a significant effect of cognitive fatigue on the number of relapses. They also demonstrated a significant higher loss of brain volume. In our view, the fact that changes in the lateral ventricle volume and the diffusivity of the corpus callosum did not reach statistical significance is only due to the reduction of the sample size by focusing only on relapsing-remitting patients.

In our view, the fact that changes in the lateral ventricle volume and the diffusivity of the corpus callosum did not reach statistical significance focusing only on relapsing remitting patients is only due to the reduction of the sample size.

Our study clearly has some limitations. First, the number of patients and controls was relatively small, but for the type of MRI analyses used it is still within the range proposed by Harrison et al. (14) Second, the group of MS patients was rather heterogeneous, encompassing patients with relapsing remitting and progressive disease courses, with a large age range and with and without immunomodulatory treatments. Still, we believe it is noteworthy that MS patients with cognitive fatigue at t1 showed increased neurodegeneration and suffered more relapses during the following 17 months as implied by our model for fatigue (2). The relevance of fatigue for changing immunomodulatory medication in MS has recently become accepted in a consensus paper (29). Our study, if replicated, underlines this step toward one additional red flag for reconsidering the treatment strategy.

## Author Contributions

CS: Analysis and interpretation of data for the work, drafting the work, final approval of the version and agreement to be accountable for all aspects of the work. PE: help on writing the manuscript, KH: assessment of study participants, JK: help on MRI data assessment and analysis, AK: help on planning and sponsoring the study, HH: help on planning and executing the study, help on statistical analysis and manuscript writing.

## Conflict of Interest Statement

The authors confirm that there are no conflicts of interest and patent holdings related to this article. Novartis Inc. sponsored this research.

## Abbreviations

BDI, Beck’s Depression Inventory; DTI, diffusion tensor imaging; FSMC, Fatigue Scale for Motor and Cognitive Functions; FSS, Fatigue Severity Scale; MSFC, Multiple Sclerosis Functional Composite; NEX, Number of excitations; PASAT, Paced Auditory Serial Addition Test.
